# One the genus *Tocama* Reitter (Coleoptera, Scarabaeidae, Melolonthinae), with descriptions of two new species from Indochina

**DOI:** 10.3897/zookeys.177.2482

**Published:** 2012-03-23

**Authors:** Chun-Lin Li, Chuan-Chan Wang, Denis Keith, Ping-Shih Yang

**Affiliations:** 1 Department of Entomology, National Taiwan University, Taipei City 106, Taiwan, ROC; 2 Department of Life Science, Fu Jen Catholic University, Hsinchuang, New Taipei City 24205, Taiwan, ROC; 3 Muséum d’Histoire Naturelle et de Préhistoire, 5 bis, boulevard de la Courtille, F - 28000 Chartres, France

**Keywords:** *Tocama*, new species, new synonym, new combination, Scarabaeidae, Melolonthinae, Indochina

## Abstract

Two new species of the Oriental scarab genus *Tocama* Reitter, 1902, *Tocama laosensis* **sp. n.** and *Tocama procera* **sp. n.**, are described from Indochina with diagnoses, distributions, remarks and illustrations. A key to the species of the genus is provided with a checklist with several nomenclatural changes: *Hoplosternus tonkinensis* Moser, 1913 is transferred to *Tocama*; *Hoplosternus pygidialis* Moser, 1915 **syn. n.**, *Tocama atra atra* Keith, 2006 **syn. n.** and *Tocama atra reichenbachi* Keith, 2007 **syn. n.** = *Tocama tonkinensis* (Moser).

## Introduction

The Oriental genus *Tocama* Reitter, 1902 is a small scarab beetle group that includes seven species, two of which are newly described here. Species of *Tocama* are distributed in southeastern Asia, eastern and southern China, and Taiwan. *Tocama* species are 14.0–30.5 mm in length, castaneous to blackish brown beetles that are dorsally covered with dense, tiny, brownish grey or yellowish brown, scale-like setae. The genus *Tocama* was first proposed by Reitter (1902) based on a single species, *Melolontha rubiginosa* Fairmaire, 1889, as a subgenus of the genus *Melolontha* Fabricius, 1775. In his paper, however, Reitter merely described the color of dorsal vestiture and fine punctation and provided a simple illustration of the parameres of the male genitalia. The male genitalia of *Melolontha rubiginosa* are strongly asymmetrical with a bundle of curled, robust setae at the apices; these constitute a set of autapomorphic characters within the tribe Melolonthini. Accordingly, Kryzhanovskij (1978) elevated *Tocama* to the generic level. Keith (2006, 2007) subsequently described two new species and one new subspecies on the grounds of genital characters. Li et al. (2010) recently transferred two more species from the genus *Melolontha* and identified two *Melolontha* species with two *Tocama* species, respectively, and thus proposed two synonymies. Based on the examination of all known *Tocama* species, we include additional characters of the clypeus, pronotum, and patterns of setae and punctation along the descriptions of two new species from Indochina. A key to the males of the genus is also provided.

## Material and methods

Specimens examined in this study were borrowed from the institutions (name of curators in acknowledgments) listed in the section of type material or came from the first author’s personal collections.

The preparation of specimens and morphological terms used in this paper follow Li et al. (2010). Specimens and characters were examined and drawn using a Leica MZ12.5 stereomicroscope (Wetzlar, Germany) equipped with a drawing tube. The body length (BL) was measured from the apex of the clypeus to the apex of the elytra, and the body width (BW) was measured at the widest distance across the elytra. The abbreviations CL and BsL refer to the length of antennal club and basal segments, respectively, while PgW/L denote the ratio of pygidial width and length.

## Systematics

### Checklist of the genus *Tocama* Reitter

**1. *Tocama formosana* (Yu, Kobayashi & Chu, 1998)**

*Melolontha formosana* Yu, Kobayashi & Chu 1998: 206. Original combination.

**Distribution.** Taiwan.

**2. *Tocama laevipennis* (Blanchard, 1851)**

*Hoplosternus laevipennis* Blanchard 1851: 158. Original combination.

*Hoplosternus squamulatus* Frey 1969: 114. Synonymized by Li et al. (2010: 346).

**Distribution.** China; Vietnam; Laos (new country record with a female collected in Vangvien, Laos, in the first author’s collections).

**3. *Tocama laosensis* Li & Keith**, **sp. n.**

**Distribution.** Laos.

**4. *Tocama procera* Li & Keith**, **sp. n.**

**Distribution.** Vietnam.

**5. *Tocama rubiginosa* (Fairmaire, 1889)**

*Melolontha rubiginosa* Fairmaire 1889: 21. Original combination.

*Melolontha albidiventris* Fairmaire 1889: 21. Synonymized by Li et al. (2010: 346).

**Distribution.** China.

**6. *Tocama siamensis* Keith, 2006: 224**

**Distribution.** Thailand.

**7. *Tocama tonkinensis* (Moser, 1913), comb. n.**

*Hoplosternus tonkinensis* Moser 1913: 290. Original combination.

*Hoplosternus pygidialis* Moser, 1915: 589, **syn. n.**

*Tocama atra atra* Keith, 2006: 225, **syn. n.**

*Tocama atra reichenbachi* Keith, 2007: 338, **syn. n.**

**Distribution.** China; Vietnam; Laos; Myanmar.

### Tocama laosensis

Li & Keith sp. n.

urn:lsid:zoobank.org:act:6C4AB152-9560-4422-A89C-B44BF06B6D21

http://species-id.net/wiki/Tocama_laosensis

Figs 1 4 6 10–12

#### Holotype

male. LAOS: Lak 20, 22–26/VIII/1996, by local collector (deposited at Museum für Naturkunde der Humboldt Universitat (ZMHB), Berlin, Germany)

#### Type locality.

Southern Laos: Champasack province, Lak 20, 15°01’N, 105°90’E.

#### Diagnosis.

*Tocama laosensis* is distinguished from other congeners by the following combination of characters: body medium sized, thin, pronotum flat when viewed laterally (Fig. 4); head, pronotum and scutellum blackish brown, elytra dull castaneous; surface of pronotum, scutellum and elytra covered with tiny brownish grey setae, setae on vertex about 4 times length of those on pronotum and elytra; basal margin of elytra between scutellum and humeral umbone broadly ridged (Fig. 6); apical ridge of pygidium impressed and becoming concave inwardly along plane of disc; mesometasternal process vestigial; metepimeron and sides of abdominal sternites 1–6 with maculation consisting of brownish white, scale-like setae; male genitalia as in Figures 10–12.

#### Description.

**Figures 1–3.**
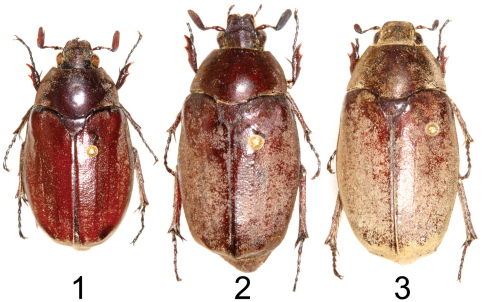
Dorsal habitus of *Tocama* spp. **1** *Tocama laosensis* sp. n., holotype male **2** *Tocama procera* sp. n., holotype male **3** *Tocama procera* sp. n., paratype female.

**Figures 4–5.**
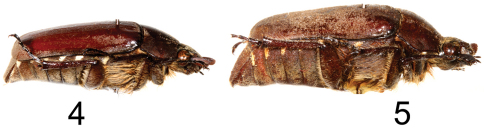
Right lateral view of *Tocama* spp. **4** *Tocama laosensis* sp. n., holotype male **5** *Tocama procera* sp. n., holotype male.

**Figures 6–7.**
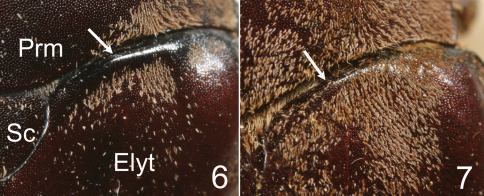
Base of right elytron of *Tocama* spp. **6** *Tocama laosensis* **7** *Tocama procera*. Prm, pronotum; Sc, scutellum; Elyt, elytron.

**Figures 8–9.**
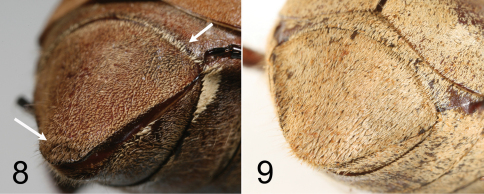
Right oblique view of pygidium of *Tocama procera* **8** holotype male. **9** paratype female.

**Figures 10–15.**
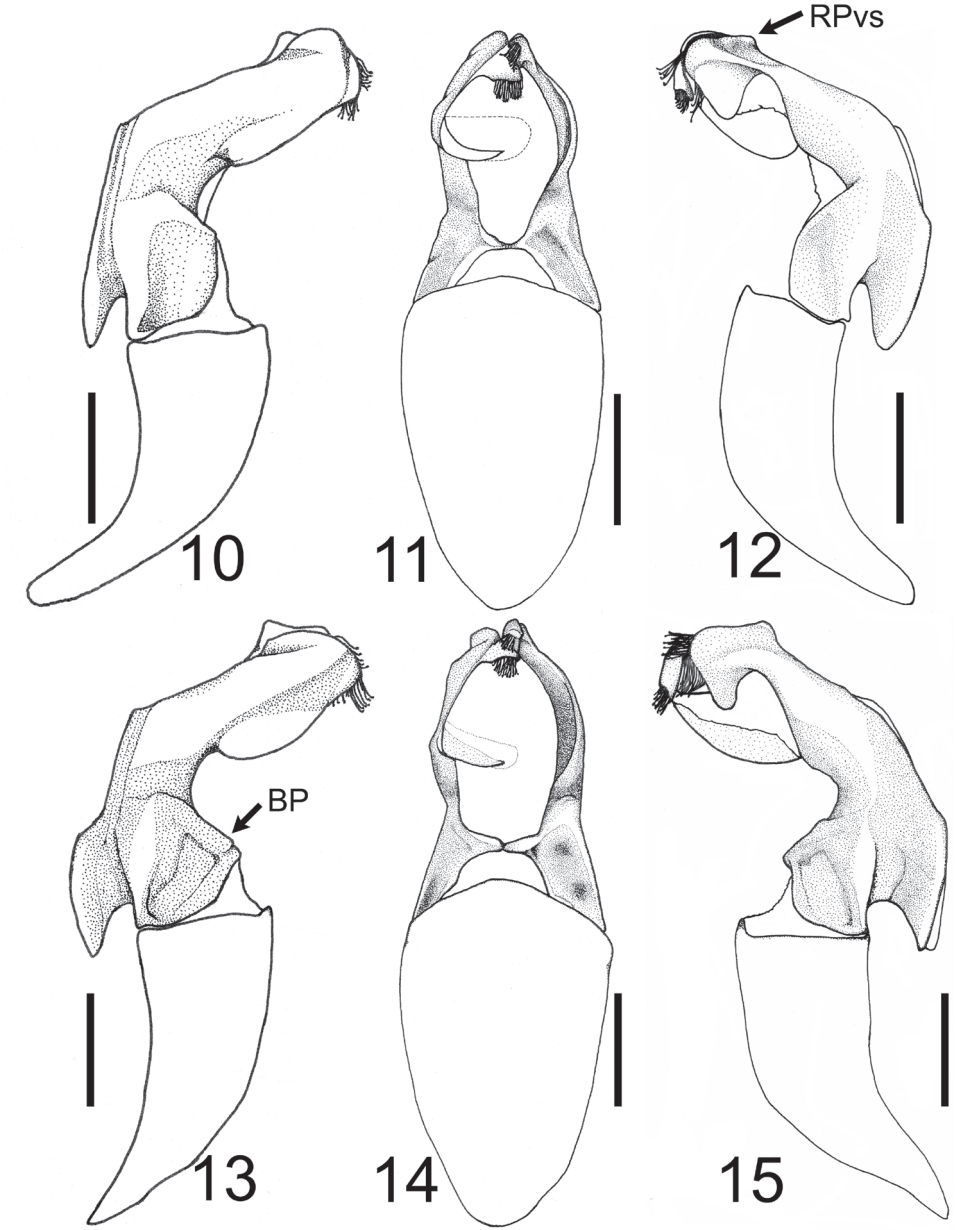
Male genitalia of *Tocama* spp. **10–12** *Tocama laosensis* sp. n. **13-15** *Tocama procera* sp. n. BP, base of parameres; RPvs, ventroapical swelling of right paramere. Scale bar = 1.5 mm.

Males(Figs 1, 4): BL: 20.0 mm; BW: 12.0 mm; CL/BL=1.1; PgW/L= 1.32. Body thin, pronotum flat when viewed laterally (Fig. 4). Head, antennae, pronotum, scutellum and venter blackish brown; tarsomeres black; elytra dull castaneous; dorsal surface of body covered with minute, brownish grey setae. *Head*: Surface densely and coarsely punctate, each puncture with a seta, setae on clypeus thinner and shorter than those on vertex. Clypeus rectangular, bordered, with center apex emarginated; vertex slightly convex with setae about 4 times length of those on pronotum and elytra. Antennal club straight, subequal in length to basal segments. Labrum strongly bilobed at middle, symmetrical, each lobe rounded apically. Mentum with anterior margin moderately bilobed, surface sparsely setigerous, setae moderately long. Maxillary palpi short, apical palpomere about half length of antennomere 3. *Pronotum*: wider than long, widest at base, depressed when viewed laterally (Fig. 4); lateral margins well bordered, weakly developed anterior to scutellum; surface densely, evenly punctuate; punctures fine, each with a scale-like, tiny seta about 3 times length of diameter of puncture. Scutellum semicircular, surface with punctures and setae similar to those on pronotum. *Elytron*: Widest at middle; surface rugose with 4 weakly developed, punctate costae, costae 1–3 (starting from suture) complete, costa 4 vestigial; overall punctures and setae same as those on disc of pronotum; basal margin of elytra between scutellum and humeral umbone broadly ridged (Fig. 6). *Propygidium*: Surface densely punctate, punctures setigerous, setae similar to those on disc of pronotum with a row of more robust setae along apical margin. *Pygidium*: Lateral margins narrowly flattened. Surface densely punctuate; punctures setigerous, setae scale-like, longer and more robust than those on pronotum, sparsely intermixed with hair-like, long setae (about 3–12 times longer than scale-like setae); pygidial apex truncate and concave inwardly along plane of disc (see Fig. 8 for *Tocama procera*), apical margin weakly quadrate. *Venter*: Prosternal process feebly protruding, apex not reaching base of protrochanter. Mesometasternal process vestigial. Metepisternum densely covered with hair-like setae. Metepimeron and sides of abdominal sternites 1–6 with maculation of brownish white, scale-like setae. Middle of abdominal sternites 1–4 almost impunctate, sides densely punctuate; punctures setigerous, setae fine, scale-like, intermixed with hair-like setae that are 3–15 times longer. *Legs*: Protibia tridentate with basal tooth weakly developed. Pro- and mesofemora flattened, surface hairy; hind femora stout, broad, surface clothed with much shorter, robust setae than those of pro- and mesofemora. Mesotibia with 2 apical spurs equal in length. Metatibia with dorsal apical spur reaching to middle of metatarsomere 2; ventral apical spur of metatibia subequal in length to metatarsomere 1. *Parameres*: In lateral view (Figs 10, 12), base of parameres (BP) constricted, ventroapical swelling of right paramere (RPvs) weakly developed. Middle of lateral margin smooth when viewed dorsally (Fig. 11).

Female unknown.

#### Etymology.

The species epithet is derived from the name of Laos, wherefrom it is described. This is the first *Tocama* species from this country and is probably endemic to it.

#### Remarks.

*Tocama laosensis* is known from a single male specimen collected by a local collector, and it lacks further information. However, the type locality of the species is the southernmost distribution for the genus so far.

### Tocama procera

Li & Keith sp. n.

urn:lsid:zoobank.org:act:D919D097-59ED-48B3-8A01-45725D1EB6FD

http://species-id.net/wiki/Tocama_procera

Figs 2, 3 5 7 9 13–15 17

#### Holotype

♂. VIETNAM: Vinh Phu//Tam Dao//19–21/V/1995//coll. M. Satô (deposited at the Natural History Museum (BMNH), London, U. K.).

#### Paratypes.

3 ♂♂ 1 ♀. VIETNAM: Lao Cai province//Sa Pa//May 3–28, 1993//N. Katsura leg. (1♂, deposited in the first author’s collection, CLLI); same locality, 11–14/V/1995// coll. M. Satô (1 ♀, CLLI); same locality, 14. V. 2000// S. Nomura leg. (1♂, deposited at the National Science Museum (NSMT), Tokyo, Japan); Tonkin// Env. de Hoa-Binh// J.Laisi_1902// ex Museo Oberthur (1♂, deposited at the Institut Royal des Sciences Naturelles de Belgique (ISNB), Bruxelles, Belgium)

#### Type locality.

Northern Vietnam: Vinh Phu province, Tam Dao, 21°46'N, 105°65'E.

#### Diagnosis.

Based upon the shared characters of the presence of metepimeral maculation (as well as lacking metepisternal maculation (Fig. 17)), apex truncate of the pygydium and shape of the parameres, *Tocama procera* most closely resembles *Tocama laosensis*. *Tocama procera* differs from *Tocama laosensis* by the thickness of the pronotum when viewed laterally (flat in *Tocama laosensis* (Fig. 4), convex in *Tocama procera* (Fig. 5)), the shape of the anterior margin of the mentum (moderately bilobed in *Tocama laosensis*, straight in *Tocama procera*), length of setae on the anterior and lateral margins of the pronotum to length of those on the disc (2–4 times longer in *Tocama laosensis*, 4–8 times longer in *Tocama procera*), and the form of the basal margin of the elytra between the scutellum and humeral umbone (broadly ridged in *Tocama laosensis* (Fig. 6), feebly ridged in *Tocama procera* (Fig. 7)).

#### Description.

Males(Figs 2, 5): BL: 20.3–22.9 mm; BW: 11.4–11.8 mm; CL/BsL=1.16–1.18; PgW/L= 1.25–1.33. Body thick, pronotum convex when viewed laterally (Fig. 5). Head, antennae, pronotum, scutellum and venter of body blackish brown, tarsomeres black, elytra dull castaneous, dorsal surface of body covered with minute, brownish grey setae. *Head*: Surface densely, coarsely punctate, each puncture with a seta, setae on clypeus thinner and shorter than those on vertex. Clypeus rectangular, bordered, emarginated apically; vertex moderately convex, covered with slender, hair-like and robust, scale-like setae, setae becoming longer laterally. Antennal club straight, subequal in length to basal segments. Labrum strongly bilobed at middle, symmetrical, each lobe rounded apically. Mentum with anterior margin straight, surface sparsely setigerous, setae moderately long. Maxillary palpi short, apical palpomere about 2/3 length of antennomere 3. *Pronotum*: Wider than long, widest at base, moderately convex when viewed laterally (Fig. 5); lateral margins well bordered but weakly developed anterior to scutellum; disc densely, evenly punctuate; punctures setigerous, fine, each with small, scale-like seta about 3 times longer than diameter of puncture, anterior and lateral margins with long, hair-like setae sparsely distributed, about 4–8 times longer than those on disc. Scutellum semicircular, surface with punctures and setae similar to those on pronotum. *Elytron*: Widest at middle. Surface rugose with 4 poorly developed, punctate costae between suture and humerus, sutural costa (as costa 1) and costae 2–3 complete, costa 4 vestigial; punctures and setae same as those on disc of pronotum; basal margin of elytra between scutellum and humeral umbone feebly ridged (Fig. 7). *Propygidium*: Surface densely punctuate, punctures setigerous; setae similar to those on disc of pronotum with a row of whitish, more robust setae along apical margin (Fig. 8). *Pygidium*: Lateral margins narrowly flattened. Surface densely punctate, puncture setigerous; setae scale-like, longer and more robust than those on pronotum, sparsely intermixed with long, hair-like setae (about 3–12 times longer than scale-like setae); pygidial apex truncate and concave inwardly along plane of disc (Fig. 8). *Venter*: Prosternal process moderately protruding, reaching base of protrochanter. Mesometasternal process feebly protruding. Metepisternum densely with hair-like setae (Fig. 17). Metepimeron and sides of abdominal sternites 1–6 with maculation consisting of brownish white, scale-like setae. Middle of abdominal sternites 1–4 almost impunctate with sides densely punctate, punctures setigerous, setae fine, scale-like, sparsely intermixed with hair-like setae 3–15 times longer. *Legs*: Protibia tridentate with basal tooth weakly developed. Pro- and mesofemora flattened, surface setose; hind femur more stout, surface clothed with much shorter, robust setae than those on pro- and mesofemora. Mesotibia with 2 apical spurs equal in length. Metatibia with dorso-apical spur reaching middle of metatarsomere 2; ventral apical spur of metatibia subequal in length to metatarsomere 1. *Parameres*: In lateral view, base of parameres (BP) strongly broadened, ventroapical swelling of right paramere (RPvs) distinctly developed (Figs 13, 15). Middle of lateral margin convex when viewed dorsally (Fig. 14).

**Figures 16–17.**
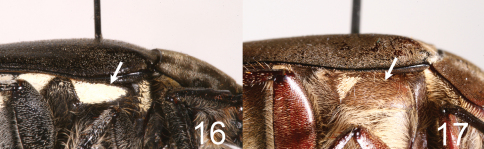
Right metepisternum of *Tocama* spp. **16** *Tocama tonkinensis* **17** *Tocama procera* sp. n.

Female (Fig. 3). BL: 22.0 mm; BW: 11.0 mm; CL/BsL= 0.63; PgW/L= 1.41. Similar to male except for setae being yellowish brown (brownish grey in males), more robust and denser on elytral apical depressed area, propygidum, pygidium and abdominal sternites; setae on metepisternum and metepimeron scale-like, forming maculation (setae long, hair-like in males, not forming maculation); antennal club 6-segmented (seven segments in males), compact and shorter than basal segments; form of pronotum enlarged anteroposteriorly; propygidium with scale-like setae; surface of pygidium flat, apex rounded, not truncate (Fig. 9); abdomen stouter, with scale-like setae everywhere except central part.

#### Etymology.

The species epithet, *procera*, is Latin for “tall” which refers to the relatively convex pronotum when viewed laterally. The name is a feminine adjective.

#### Remarks.

Based on the collecting records of the type series, *Tocama procera* is geographically restricted to the mid-elevated (1,000–1,600m) montane areas of northern Vietnam where it occurs sympatrically with *Tocama tonkinensis*. However, *Tocama tonkinensis* has the broadest geographical range within the genus, ranging throughout northern Indochina (Vietnam, Laos, and Myanmar) northward to southern China (Yunnan, Guangxi, Guizhou, and Hunan).

### Tocama tonkinensis

(Moser, 1913) comb. n.

Hoplosternus tonkinensis Moser 1913: 290. Original combination
Hoplosternus pygidialis Moser, 1915: 589, **syn. n.**
Tocama atra atra Keith, 2006: 225, **syn. n.**
Tocama atra reichenbachi Keith, 2007: 338, **syn. n.**

#### Distribution.

China; Vietnam; Laos (new country record with a male collected in Mt. Phu Pan, Houaphan province, Laos, in the first author’s collections); Myanmar (new country record with two females collected nearby Putao, Kachin state, Myanmar, in the first author’s collections)

#### Remarks.

Moser (1913) described *Hoplosternus tonkinensis* based from a female (labeled as “Tonkin [presently northern Vietnam] Montes Manson April. Mai 2–3000’ H Fruhstorfer (printed) // Hoplosternus tonkinensis Type Mos (handwritten)”, currently deposited at Museum für Naturkunde der Humboldt Universitat (ZMHB)), Berlin, Germany). Subsequently, he described *Hoplosternus pygidialis* Moser, 1915, from a male (labeled as “Kiautschou [Guizhou Province] China (printed)// Hoplosternus pygidialis Type Mos (handwritten), deposited at ZMHB). We examined both type specimens and found that they share series of characters of pronotum shape, punctuation, setation, and male genitalia shape; these characters clearly indicate that the specimens are members of *Tocama*. Moreover, having examined large number of *Tocama tonkinensis* specimens (45 males and 43 females) from Indochina and China, we found that the sexual dimorphism of the species is particularly distinct in the shape of the pygidium, which might be the reason of separation for the previous two species by Julius Moser. We conclude that these two type specimens are conspecific.

Additionally, the intraspecific variation of *Tocama tonkinensis* is significant in body color. There are two main forms of body color, black and castaneous, that led Keith (2006, 2007) to separation of *Tocama atra atra* (black form) and *Tocama atra reichenbachi* (castaneous form). We consider that they are all within the variability of *Tocama tonkinensis* based on the examination of the type series of *Tocama atra atra* and *Tocama atra reichenbachi* (see also Figs 5–6 in Keith (2006) for the shape of male genitalia). Accordingly, we herein transfer *Hoplosternus tonkinensis* to *Tocama* and synonymize the names *Hoplosternus pygidialis*, *Tocama atra atra* and *Tocama atra reichenbachi* with *Tocama tonkinensis*.

### Key to the males of *Tocama* species

**Table:** 

1	Body length≤17.9 mm; mesometasternal process absent	2
–	Body length≥19.6 mm; mesometasternal process vestigial to significantly developed, varying in size and shape	3
2	Disc of pronotum evenly covered with short setae; surface of metepimeron covered with white, scale-like setae	*Tocama rubiginosa* (Fairmaire)
–	Disc of pronotum covered with long and short setae centrally, with long, hairy-like setae laterally; surface of metepimeron covered with brownish white, hair-like setae	*Tocama formosana* (Yu, Kobayashi & Chu)
3	Elytral costae visible, varying in development; metepimeron and sides of abdominal sternites with maculation consisting of overlapping, white, scale-like setae	4
–	Elytral costae entirely absent on disc, sutural and epipleural costae vestigial; metepimeron and sides of abdominal sternites without maculation; body length≥24.7 mm	*Tocama laevipennis* (Blanchard)
4	Metepisternum with maculation (Fig. 16)	5
–	Metepisternum without maculation (Fig. 17)	6
5	Antennal club slightly curved outwardly, longer than stem (CL/BsL=1.8–2.0); elytral costae feebly developed with surface punctate; mesometasternal process long and sharp, apex reaching anterior margin of procoxae; tip of pygidium broadly concave inwardly	*Tocama tonkinensis* (Moser)
–	Antennal club straight, subequal in length to stem (CL/BsL≈1.1); elytral costae strongly raised, surface of sutural costa and costae 1–2 impunctate; mesometasternal process broadly truncate, apex reaching anterior margin of mesocoxae; pygidium tapering apically	*Tocama siamensis* Keith
6	Body slender, pronotum flat when viewed laterally (Fig. 4); anterior and lateral margins of pronotum with variably long setae, setae about 2–4 times length of those on disc; basal margin of elytra between scutellum and humeral umbone broadly ridged (Fig. 6)	*Tocama laosensis*, new species
–	Body robust, pronotum convex when viewed laterally (Fig. 5); anterior and lateral margins of pronotum with variably long setae, setae about 4–8 times length of those on disc; basal margin of elytra between scutellum and humeral umbone feebly ridged (Fig. 7)	*Tocama procera*, sp. n.

## Supplementary Material

XML Treatment for Tocama laosensis

XML Treatment for Tocama procera

XML Treatment for Tocama tonkinensis
